# Association of Thyrotropin Suppression With Survival Outcomes in Patients With Intermediate- and High-Risk Differentiated Thyroid Cancer

**DOI:** 10.1001/jamanetworkopen.2018.7754

**Published:** 2019-02-01

**Authors:** Joanna Klubo-Gwiezdzinska, Sungyoung Auh, Marvin Gershengorn, Brianna Daley, Athanasios Bikas, Kenneth Burman, Leonard Wartofsky, Mark Urken, Eliza Dewey, Robert Smallridge, Ana-Maria Chindris, Electron Kebebew

**Affiliations:** 1 National Institute of Diabetes and Digestive and Kidney Diseases, National Institutes of Health, Bethesda, Maryland; 2 Endocrine Section, Medstar Washington Hospital Center, Washington, DC; 3 Institute of Head, Neck and Thyroid Cancer, Mount Sinai Beth Israel Medical Center, New York, New York; 4 Department of Endocrinology and Cancer Center, Mayo Clinic, Jacksonville, Florida; 5 Department of Surgery and Stanford Cancer Institute, Stanford University, Stanford, California

## Abstract

**Importance:**

Suppression of thyrotropin (often referred to as *thyroid-stimulating hormone*, or *TSH*) with levothyroxine used in management of intermediate- and high-risk differentiated thyroid cancer (DTC) to reduce the likelihood of progression and death is based on conflicting evidence.

**Objective:**

To examine a cohort of patients with intermediate- and high-risk DTC to assess the association of thyrotropin suppression with progression-free survival (PFS) and overall survival.

**Design, Setting, and Participants:**

This cohort study used a multicenter database analysis including patients from tertiary referral centers and local clinics followed up for a mean (SD) of 7.2 (5.8) years. Patients with DTC treated uniformly with total thyroidectomy and radioactive iodine between January 1, 1979, and March 1, 2015, were included. Among the 1012 patients, 145 patients were excluded due to the lack of longitudinal thyrotropin measurements.

**Exposures:**

Levothyroxine therapy to target thyrotropin suppression with dose adjustments based on changing thyrotropin goal.

**Main Outcomes and Measures:**

The primary outcome measures were overall survival and PFS. A Cox proportional hazards model was used to assess the contribution of age, sex, tumor size, histology, and lymph node and distant metastases at landmarks 1.5, 3.0, and 5.0 years. The patients were divided into 3 groups based on mean thyrotropin score before each landmark: (1) suppressed thyrotropin, (2) moderately suppressed or low-normal thyrotropin, and (3) low-normal or elevated thyrotropin.

**Results:**

Among 867 patients (557 [64.2%] female; mean [SD] age, 48.5 [16.5] years) treated with a median (range) cumulative dose of 151 (30-1600) mCi radioactive iodine, disease progression was observed in 293 patients (33.8%), and 34 patients (3.9%) died; thus, the study was underpowered in death events. Thyrotropin suppression was not associated with improved PFS at landmarks 1.5 (*P* = .41), 3.0 (*P* = .51), and 5.0 (*P* = .64) years. At 1.5 and 3.0 years, older age (hazard ratio [HR], 1.06; 95% CI, 1.03-1.08 and HR, 1.05; 95% CI, 1.01-1.08, respectively), lateral neck lymph node metastases (HR, 4.64; 95% CI, 2.00-10.70 and HR, 4.02; 95% CI, 1.56-10.40, respectively), and distant metastases (HR, 7.54; 95% CI, 3.46-16.50 and HR, 7.10; 95% CI, 2.77-18.20, respectively) were independently associated with subsequent time to progression, while at 5.0 years, PFS was shorter for patients with lateral neck lymph node metastases (HR, 3.70; 95% CI, 1.16-11.90) and poorly differentiated histology (HR, 71.80; 95% CI, 9.80-526.00).

**Conclusions and Relevance:**

Patients with intermediate- and high-risk DTC might not benefit from thyrotropin suppression. This study provides the justification for a randomized trial.

## Key Points

**Question:**

Is thyrotropin suppression associated with better outcomes in patients with intermediate- and high-risk differentiated thyroid cancer?

**Findings:**

In this cohort study including 867 patients with intermediate- and high-risk differentiated thyroid cancer followed up for a mean (SD) of 7.2 (5.8) years, thyrotropin suppression was not associated with improved progression-free survival or overall survival.

**Meaning:**

Patients with intermediate- and high-risk differentiated thyroid cancer might not benefit from thyrotropin suppression.

## Teaser

This cohort study investigates the association between thyrotropin suppression and survival outcomes in patients with intermediate- and high-risk differentiated thyroid cancer.

## Introduction

Currently accepted therapy for patients with intermediate- or high-risk differentiated thyroid cancer (DTC) is surgery followed by radioactive iodine (RAI) therapy. Radioactive iodine therapy is performed under either endogenous or exogenous stimulation of thyrotropin (often referred to as *thyroid-stimulating hormone*, or *TSH*), aimed at achieving a thyrotropin level exceeding 30 mIU/L.^1^ Thyrotropin stimulation leads to overexpression of thyroid differentiation genes such as sodium-iodide symporter, resulting in increased RAI uptake, enhancing the tumoricidal effect.^1,2,3^ The long-term management of DTC includes levothyroxine therapy aimed not only at appropriate physiologic thyroid hormone replacement in thyroidectomized patients, but also at suppressing thyrotropin release from the pituitary gland by supraphysiologic levothyroxine doses. A recommended thyrotropin suppression goal in patients with locally advanced or metastatic DTC is less than 0.1 mIU/L as some preclinical evidence suggests that thyrotropin is a growth stimulus for DTC cells.^4^

The growth regulatory effects of thyrotropin in in vitro and in vivo models remain controversial. While some studies suggest a biphasic growth response curve with thyrotropin being a differentiation stimulus at physiologic concentrations, and growth stimulus at higher concentrations,^5^ other studies show thyrotropin alone does not stimulate proliferation but, rather, requires the presence of insulinlike growth factor 1 or insulin signaling to stimulate cancer growth.^4^ In addition, in vivo transgenic mouse models indicate that thyrotropin receptor signaling is involved in the genesis of goiters, but not sufficient to induce carcinogenesis, and thyrotropin suppression did not revert disease progression in a metastatic mouse model.^6,7^ Moreover, thyrotropin receptor expression is significantly lower in human thyroid cancer tissue, specifically tissue harboring a *BRAF *V600E mutation, compared with normal tissue.^8^

Clinical data evaluating the relationship between thyrotropin suppression and long-term DTC outcomes such as survival are equivocal^9,10,11,12,13,14,15,16,17,18,19,20,21,22,23,24,25,26,27,28,29^ (eTable 1 in the Supplement). Several studies have uniformly documented that thyrotropin suppression is not necessary in patients with low-risk DTC.^1,15,20,21,22,23,24,25,26,27^ However, there is significant discrepancy in the role of thyrotropin suppression in patients with intermediate- or high-risk DTC. Nonetheless, the current American Thyroid Association (ATA) guidelines recommend levothyroxine therapy with doses adjusted to achieve thyrotropin suppression below 0.1 mIU/L in patients with high-risk DTC, moderate thyrotropin suppression to a goal of 0.1 to 0.5 mIU/L in patients with intermediate-risk DTC, and, for patients with low-risk DTC, a thyrotropin goal of 0.5 to 2 mU/L.^1^ Another factor that complicates the evaluation of the role of thyrotropin suppression over time is that the thyrotropin goal can be modified based on response to therapy. Patients with structurally or biochemically incomplete response continue to receive levothyroxine doses aimed at full thyrotropin suppression of less than 0.1 mIU/L; patients with indeterminate response have a thyrotropin goal of 0.1 to 0.5 mIU/L; and patients with excellent response to therapy have a thyrotropin goal liberalized to low-normal values of 0.5 to 2.0 mIU/L.^1^ These recommendations are based predominately on retrospective studies with limited numbers of patients and thyrotropin measurements.

These controversies formed the rationale for our study, which was aimed at assessing the association between thyrotropin suppression reflected by measurement of thyrotropin levels over time and overall survival (OS) and progression-free survival (PFS) in patients with intermediate- or high-risk DTC.

## Methods

### Design

We performed a retrospective cohort study of patients with ATA intermediate- or high-risk thyroid cancer who underwent initial treatment consisting of total thyroidectomy and therapy with RAI between January 1, 1979, and March 1, 2015, and were followed up through April 13, 2017. All patients were subsequently treated with levothyroxine with a goal to longitudinally suppress thyrotropin levels to values less than 0.1 mIU/L. The associations between the degree of thyrotropin suppression over time and PFS and OS were examined. The degree of thyrotropin suppression in each patient varied during follow-up. Accordingly, a previously reported and validated thyrotropin scoring system was used to measure thyrotropin levels on follow-up.^21,26^ To account for data analysis with time-varying covariate, a landmark analysis was performed. This study followed the Strengthening the Reporting of Observational Studies in Epidemiology (STROBE) reporting guideline for cohort studies.

### Participants

We obtained the multi-institutional review board approval to perform the study based on analysis of the Thyroid Cancer Care Collaborative, National Institutes of Health, Medstar, and Mayo Clinic, Florida, databases. Requirements for patient informed consent were waived by the institutional review board; however, all patients participating at the National Institutes of Health provided written informed consent per institutional policy. Participants were patients with DTC who fulfilled the following inclusion criteria:

1. Intermediate risk patients presenting with aggressive histology (columnar/tall cell, insular variant, Hurthle cell), vascular invasion, tumor size T3 or T2 with known *BRAF* V600E mutation, or clinical lymph node involvement N1; or high-risk patients presenting with either tumor size T4 with gross extrathyroidal extension or large lymph node metastases greater than 3 cm or distant metastases,
2. Patients treated uniformly with total thyroidectomy with or without lymph node dissection, as clinically indicated, and RAI between January 1979 and March 2015, and
3. Patients with available follow-up data for thyrotropin level, suppressed and/or stimulated thyroglobulin (Tg), and iodine 123 or iodine 131 whole-body scans and other imaging modalities (ultrasound of neck, computed tomography of chest and neck, fludeoxyglucose F 18 positron emission tomography/computed tomography).

Exclusion criteria were low-risk DTC, ie, papillary thyroid cancer with T1 or T2N0M0, and patients without follow-up data available.

### Treatment Interventions

All patients were uniformly treated with thyroidectomy and RAI. Following thyroidectomy, all patients were treated with levothyroxine with an initial goal to longitudinally suppress thyrotropin levels to values less than 0.1 mIU/L. Levothyroxine therapy efficacy was assessed by third-generation thyrotropin assays with functional sensitivities of at least 0.1 mIU/L performed at each institution’s clinical laboratory. Best overall response to treatment was based on suppressed and/or stimulated Tg levels, whole-body scans, and other imaging modalities performed during follow-up visits occurring a mean (SD) of every 12 (6) months. Best overall response was assessed based on the ATA definition: “(1) excellent response (ER)—negative imaging, suppressed Tg <0.2 ng/mL or stimulated Tg <1 ng/mL; (2) biochemically incomplete response (BIR)—negative imaging, suppressed Tg >1 ng/mL, stimulated Tg >10 ng/mL or rising anti-Tg [antibody] levels; (3) structurally incomplete response (SIR)—structural or functional evidence of disease with any Tg level+/−Tg [antibody]; (4) indeterminate response (IR)—nonspecific imaging findings, faint uptake in thyroid bed on RAI scanning, nonstimulated Tg detectable but <1 ng/mL, stimulated Tg detectable but <10 ng/mL or Tg antibodies stable or declining in the absence of structural or functional disease.”^1^

Response to treatment was an important variable that could have led to a change in the degree of thyrotropin suppression over time, as patients with SIR or BIR should have continued to receive levothyroxine doses aimed at full thyrotropin suppression of less than 0.1 mIU/L, but patients with IR could have had levothyroxine adjusted to a thyrotropin goal of 0.1 to 0.5 mIU/L; for patients with ER, the thyrotropin goal could have been liberalized to 0.5 to 2 mIU/L.^1^ Moreover, the degree of thyrotropin suppression in each patient was variable during follow-up as a result of (1) necessity to stimulate thyrotropin to greater than 30 mIU/L repeatedly during the follow-up to perform either diagnostic studies and/or repeated therapies with RAI, (2) optimization and adjustment of therapeutic dose of levothyroxine over time, and (3) patient’s compliance.

### Primary Outcomes

The primary outcome measures were OS and PFS. We calculated OS from the date of initial thyroidectomy until the date of death. We calculated PFS from the date of initial thyroidectomy to the date of the first evidence of structural disease progression as defined per Response Evaluation Criteria in Solid Tumors (RECIST) 1.1 criteria.^30^ Patients who did not experience these events were censored at the last follow-up visit. The associations between the degree of thyrotropin suppression over time and PFS and OS were examined.

### Statistical Analysis

#### Thyrotropin Measurement

We needed to determine the longitudinal average of the thyrotropin values for each patient. To account for the significant variability in thyrotropin levels ranging between less than 0.1 mIU/L (suppressed) and greater than 30 mIU/L (stimulated), a previously reported thyrotropin scoring system was implemented, with a score of 1 indicating thyrotropin level aggressively suppressed to less than 0.1 mIU/L; a score of 2, thyrotropin level moderately suppressed to 0.1 to less than 0.5 mIU/L; a score of 3, low-normal thyrotropin level of 0.5 to less than 2 mIU/L; and a score of 4, elevated thyrotropin level of greater than 2 mIU/L.^12,21,26^ The implementation of a scoring system was necessary, as the arithmetic average thyrotropin level during follow-up would have falsely skewed the results to the right (eTable 2 in the Supplement).

To account for data analysis with time-varying covariate, a landmark analysis was performed at landmarks 1.5, 3.0, and 5.0 years. The 1.5-year landmark was selected because the first analysis of treatment response occurs within a mean (SD) of 12 (6) months following thyroidectomy and RAI, while the landmarks of 3.0 and 5.0 years were chosen to comply with general reporting rules for oncology studies. At each landmark, patients were divided into 3 groups based on mean categorized prelandmark thyrotropin scores obtained by using thyrotropin score measured between the date of thyroidectomy and the landmark date: (1) thyrotropin-S, indicating mean thyrotropin score 1 to less than 2, ie, suppressed thyrotropin; (2) thyrotropin-ML, indicating mean thyrotropin score 2 to less than 3, ie, moderately suppressed to low-normal thyrotropin; and (3) thyrotropin-LE, indicating mean thyrotropin score 3 to 4 with low-normal to elevated thyrotropin.

#### Analysis

The baseline clinical characteristics of the study population were summarized using means with standard deviations, medians with interquartile ranges, or proportions.

To test the association between the thyrotropin group and survival outcomes (PFS and OS), an unadjusted Kaplan-Meier survival analysis was performed. To account for the factors that have strong clinical evidence of being confounding variables, age (continuous), sex (male vs female), tumor size (continuous), histology (different histology types vs classic papillary thyroid cancer), presence of central and lateral neck lymph node metastases (vs no lymph node metastases) and distant metastases (vs no distant metastases), and gross extrathyroidal extension (vs no extrathyroidal extension), a Cox proportional hazards model was used. The survival analysis was started from each landmark. We found that there was insufficient evidence of nonproportionality for the evaluation of PFS and OS at each landmark and, thus, assumed that the proportionality assumption was met (eTable 3 in the Supplement). Estimated hazard ratios (HRs) with corresponding 95% confidence intervals were reported using the final model.

All analyses were 2-tailed tests based on α = .05 and were conducted using SAS statistical software version 9.4 (SAS Institute Inc).

## Results

A total of 1012 patients met inclusion criteria. Of these participants, 867 (85.7%) had postthyroidectomy thyrotropin values and compose our study cohort (557 [64.2%] female; mean [SD] age, 48.5 [16.5] years) (eFigure 1 in the Supplement). Baseline characteristics of the study cohort and the excluded 145 patients were reasonably comparable (Table 1). All patients were treated with total thyroidectomy and RAI, with a median (range) cumulative RAI dose of 151 (30-1600) mCi. During a mean (SD) follow-up of 7.2 (5.8) years, 293 patients (33.8%) experienced disease progression and 34 patients (3.9%) died. The best overall response to treatment was excellent in 51.7% of patients, SIR in 33.6%, BIR in 9.6%, and IR in 5.1% of patients.

**Table 1.** Baseline Characteristics of the Study Cohort and Excluded Patients

Characteristic	No. (%)	
	Participants (N = 867)	Excluded Patients (n = 145)
Age at diagnosis, mean (SD), y	48.5 (16.5)	49.8 (16.7)
Female^a^	557 (64.2)	93 (64.5)
Histology of thyroid cancer		
Follicular	41 (4.7)	9 (6.2)
Hurthle cell	200 (23.1)	13 (9.0)
Poorly differentiated—insular variant	24 (2.8)	8 (5.5)
Papillary (tall cell)	73 (8.4)	10 (6.9)
Papillary (classic) with micromedullary thyroid cancer	2 (0.2)	0
Papillary (follicular variant)	96 (11.1)	17 (11.7)
Papillary (classic)	429 (49.5)	82 (56.6)
Missing information	2 (0.2)	6 (4.1)
Tumor size, mean (SD), cm	3.1 (2.2)	3.17 (2.6)
Distant metastases^b^	148 (17.1)	15 (10.3)
Pulmonary micrometastases (<1.0 cm)	122 (14.1)	3 (2.1)
Pulmonary macrometastases (≥1.0 cm)	80 (9.2)	6 (4.1)
Bone metastases	39 (4.5)	6 (4.1)
Other metastatic foci (brain, spine, kidney, skin)	28 (3.2)	12 (8.3)
Gross extrathyroidal extension^c^	281 (32.4)	76 (52.4)
Lymph node metastases^d^	442 (51.0)	65 (44.8)
Central neck lymph node metastases	416 (48.0)	65 (44.8)
Lateral neck lymph node metastases	256 (29.5)	43 (29.6)

^a^ Missing data for 2 patients.

^b^ Unknown distant metastases status at baseline for 115 participants.

^c^ Unknown gross extrathyroidal extension status for 162 participants.

^d^ Unknown lymph node metastases status for 125 participants.

The mean (SD) number of thyrotropin measurements during the follow-up was 11 (10). Mean thyrotropin scores based on thyrotropin measurements before each landmark are depicted in eTable 4 in the Supplement. Thirty percent of patients moved to a different thyrotropin group based on a change in mean thyrotropin score at each landmark.

**Figure 1.**
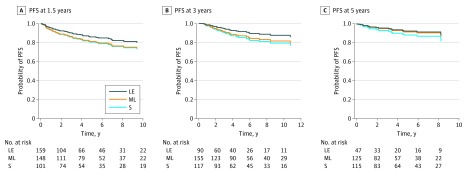
Association Between Progression-Free Survival (PFS) and Level of Thyrotropin Suppression Results were adjusted by factors independently associated with PFS: age, sex, tumor size, presence of extrathyroidal extension, lymph node metastases in central and lateral neck, and distant metastases and histology type. No difference in PFS was observed at 1.5 (A), 3.0 (B), and 5.0 (C) years. LE indicates patients with longitudinally low-normal or elevated thyrotropin (score 3-4); ML, patients with longitudinally moderately suppressed or low-normal thyrotropin (score 2 to <3); and S, patients with longitudinally suppressed thyrotropin (score 1 to <2).

We found that the degree of thyrotropin suppression was not associated with improved PFS at landmarks 1.5 (*P* = .41), 3.0 (*P* = .51), and 5.0 (*P* = .64) years (Figure 1 and Table 2; unadjusted analysis is shown in eFigure 2 in the Supplement). In patients who did not progress within the first 1.5 or 3 years, the subsequent time to progression was associated with older age (hazard ratio [HR], 1.06; 95% CI, 1.03-1.08 and HR, 1.05; 95% CI, 1.01-1.08, respectively), lateral neck lymph node metastases (HR, 4.64; 95% CI, 2.00-10.70 and HR, 4.02; 95% CI, 1.56-10.40, respectively), and distant metastases (HR, 7.54; 95% CI, 3.46-16.50 and HR, 7.10; 95% CI, 2.77-18.20, respectively) (Table 2). For patients who did not progress within the first 5 years of follow-up, the increased risk of subsequent progression was associated with presence of lateral neck lymph node metastases (HR, 3.70; 95% CI, 1.16-11.90) and poorly differentiated histology (HR, 71.80; 95% CI, 9.80-526.00) compared with classic papillary thyroid cancer (Table 2).

**Table 2.** Factors Associated With Progression-Free Survival

Factor	Hazard Ratio (95% CI)		*P* Value
**Landmark 1.5 Years**			
Thyrotropin-ML vs thyrotropin-S	0.95 (0.41-2.24)	.91	
Thyrotropin-LE vs thyrotropin-S	0.61 (0.24-1.54)	.29	
Thyrotropin-LE vs thyrotropin-ML	0.64 (0.31-1.31)	.22	
Age	1.06 (1.03-1.08)	<.001^a^	
Male sex	0.97 (0.48-1.93)	.92	
Tumor size	1.16 (0.98-1.38)	.09	
Gross extrathyroidal extension	0.87 (0.40-1.89)	.72	
Central neck LN metastases	0.93 (0.38-2.26)	.87	
Lateral neck LN metastases	4.64 (2.00-10.70)	<.001^a^	
Distant metastases	7.54 (3.46-16.50)	<.001^a^	
Poorly differentiated vs classic papillary thyroid cancer	2.36 (0.62-9.03)	.21	
**Landmark 3.0 Years**			
Thyrotropin-ML vs thyrotropin-S	0.85 (0.36-2.01)	.71	
Thyrotropin-LE vs thyrotropin-S	0.50 (0.15-1.63)	.25	
Thyrotropin-LE vs thyrotropin-ML	0.59 (0.19-1.81)	.35	
Age	1.05 (1.01-1.08)	.009^a^	
Male sex	0.92 (0.41-2.09)	.85	
Tumor size	0.89 (0.70-1.13)	.35	
Gross extrathyroidal extension	0.99 (0.38-2.61)	.99	
Central neck LN metastases	0.84 (0.33-2.15)	.72	
Lateral neck LN metastases	4.02 (1.56-10.40)	.004^a^	
Distant metastases	7.10 (2.77-18.20)	<.001^a^	
Poorly differentiated vs classic papillary thyroid cancer	3.87 (0.84-17.80)	.08	
**Landmark 5.0 Years**			
Thyrotropin-ML vs thyrotropin-S	0.65 (0.23-1.84)	.42	
Thyrotropin-LE vs thyrotropin-S	0.57 (0.12-2.82)	.49	
Thyrotropin-LE vs thyrotropin-ML	0.88 (0.17-4.53)	.87	
Age	1.02 (0.97-1.07)	.42	
Male sex	1.67 (0.62-4.46)	.31	
Tumor size	0.94 (0.70-1.27)	.69	
Gross extrathyroidal extension	1.10 (0.32-3.79)	.88	
Central neck LN metastases	0.92 (0.30-2.85)	.89	
Lateral neck LN metastases	3.70 (1.16-11.90)	.03^a^	
Distant metastases	3.57 (0.87-14.70)	.08	
Poorly differentiated vs classic papillary thyroid cancer	71.8 (9.80-526.00)	<.001^a^	

Abbreviations: LN, lymph node; thyrotropin-LE, low-normal or elevated thyrotropin; thyrotropin-ML, moderately suppressed or low-normal thyrotropin; thyrotropin-S, suppressed thyrotropin.

^a^ Statistically significant at *P* < .05.

**Figure 2.**
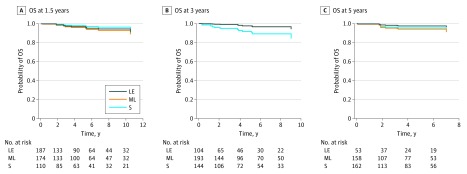
Association Between Overall Survival (OS) and Level of Thyrotropin Suppression Results were adjusted by factors independently associated with OS: age, sex, tumor size, presence of extrathyroidal extension, lymph node metastases in central and lateral neck, and distant metastases and histology type. No difference in OS was observed at 1.5 (A) and 5.0 (C) years. Improved OS was seen for nonsuppressed groups at 3.0 years (B). LE indicates patients with longitudinally low-normal or elevated thyrotropin (score 3-4); ML, patients with longitudinally moderately suppressed or low-normal thyrotropin (score 2 to <3); and S, patients with longitudinally suppressed thyrotropin (score 1 to <2).

Older age was significantly associated with shorter survival at each landmark (1.5 years: HR, 1.12; 95% CI, 1.01-1.21; 3.0 years: HR, 1.13; 95% CI, 1.03-1.24; 5.0 years: HR, 1.25; 95% CI, 1.02-1.54). In addition, presence of distant metastases was a significant factor associated with OS at landmarks 1.5 years (HR, 8.78; 95% CI, 1.19-64.60) and 3.0 years (HR, 5.33; 1.15-24.80), while presence of lateral neck lymph node metastases was associated with OS in patients who survived 3 years (HR, 28.0; 95% CI, 1.11-706.3) and 5 years (HR, 368; 95% CI, 1.19-113 464). The level of thyrotropin suppression was not associated with OS at landmarks 1.5 (*P* = .69) and 5.0 (*P* = .52) years (Figure 2, Table 3; unadjusted analysis is shown in eFigure 3 in the Supplement). In contrast, for patients who survived 3 years following thyroidectomy, individuals with nonsuppressed thyrotropin were characterized by longer subsequent OS (thyrotropin-ML vs thyrotropin-S: HR, 0.10; 95% CI, 0.01-0.82; thyrotropin-LE vs thyrotropin-S: HR, 0.10; 95% CI, 0.01-0.75). However, this analysis was limited in statistical power to detect differences in OS because our study cohort was characterized by a low number of death events. With respect to the observed median survival times, a sample size of 5252 patients would be sufficient to detect differences in OS with a power of 80%.

**Table 3.** Factors Associated With Overall Survival

Factor	Hazard Ratio (95% CI)	*P* Value	
**Landmark 1.5 Years**			
Thyrotropin-ML vs thyrotropin-S	3.26 (0.21-49.5)		.40
Thyrotropin-LE vs thyrotropin-S	2.02 (0.18-23.2)		.57
Thyrotropin-LE vs thyrotropin-ML	0.62 (0.09-4.40)		.63
Age	1.12 (1.01-1.23)		.02^a^
Male sex	0.34 (0.06-1.82)		.21
Tumor size	1.10 (0.75-1.60)		.64
Gross extrathyroidal extension	0.47 (0.06-3.90)		.48
Central neck LN metastases	1.02 (0.02-53.30)		.99
Lateral neck LN metastases	18.10 (0.31-1044.00)		.16
Distant metastases	8.78 (1.19-64.60)		.03^a^
Poorly differentiated vs classic papillary thyroid cancer	NA		NA
**Landmark 3.0 Years**			
Thyrotropin-ML vs thyrotropin-S	0.10 (0.01-0.82)		.03^a^
Thyrotropin-LE vs thyrotropin-S	0.10 (0.01-0.75)		.03^a^
Thyrotropin-LE vs thyrotropin-ML	0.99 (0.12-7.82)		.99
Age	1.13 (1.03-1.24)		.01^a^
Male sex	6.04 (0.97-37.80)		.06
Tumor size	0.84 (0.54-1.30)		.42
Gross extrathyroidal extension	0.29 (0.05-1.80)		.18
Central neck LN metastases	2.12 (0.12-39.30)		.61
Lateral neck LN metastases	28.00 (1.11-706.30)		.04^a^
Distant metastases	5.33 (1.15-24.80)		.03^a^
Poorly differentiated vs classic papillary thyroid cancer	NA		NA
**Landmark 5.0 Years**			
Thyrotropin-ML vs thyrotropin-S	3.08 (0.04-220.60)		.61
Thyrotropin-LE vs thyrotropin-S	0.11 (0.01-10.10)		.33
Thyrotropin-LE vs thyrotropin-ML	0.03 (0-13.30)		.27
Age	1.25 (1.02-1.54)		.03^a^
Male sex	1.35 (0.04-47.60)		.87
Tumor size	0.51 (0.21-1.23)		.13
Gross extrathyroidal extension	0.19 (0.002-15.70)		.46
Central neck LN metastases	74.80 (0.31-18 201.00)		.12
Lateral neck LN metastases	368.00 (1.19-113 464.00)		.04^a^
Distant metastases	2.08 (0.10-44.90)		.64
Poorly differentiated vs classic papillary thyroid cancer	NA		NA

Abbreviations: LN, lymph node; NA, not applicable; thyrotropin-LE, low-normal or elevated thyrotropin; thyrotropin-ML, moderately suppressed or low-normal thyrotropin; thyrotropin-S, suppressed thyrotropin.

^a^ Statistically significant at *P* < .05.

## Discussion

We analyzed a large cohort of patients with ATA intermediate- and high-risk DTC characterized by multiple thyrotropin measurements and followed up for more than 7 years. We found that thyrotropin suppression was not associated with improved PFS and OS. In fact, patients with suppressed thyrotropin levels who survived 3 years were characterized by shorter OS than patients whose levels were not suppressed. Because the thyrotropin level in our cohort changed over time, we performed a landmark analysis to test whether there was any time-dependent difference in the outcome. At 1.5 and 3.0 years, we found that older age, lateral neck lymph node metastases, and distant metastases were independently associated with subsequent time to progression, while for patients who did not experience progression within the first 5.0 years, the subsequent time to progression was shorter for patients with lateral neck lymph node metastases and poorly differentiated histology. Older age was significantly associated with shorter survival at each landmark. In addition, presence of distant metastases was a significant factor associated with shorter OS for patients who survived 1.5 and 3.0 years after thyroidectomy, while presence of lateral neck lymph node metastases was associated with shorter OS in patients who survived 3 and 5 years.

The results of our study challenge the current paradigm of thyrotropin suppression in patients with intermediate- or high-risk DTC. The clinical evidence of the effects of thyrotropin suppression on PFS, OS, or disease-specific survival (DSS) was derived predominantly from small retrospective cohort studies characterized by a limited number of thyrotropin measurements (eTable 1 in the Supplement). In fact, there is only 1 prospective randomized clinical open-label trial, performed in Japan, focused on the role of thyrotropin suppression in thyroid cancer recurrence rate and mortality. Although most patients enrolled in the aforementioned study were characterized by low-risk DTC, 296 patients had lymph node metastases and 50 patients had distant metastases or extrathyroidal extension.^23^ Consistent with our findings, the Japanese study did not reveal any differences in the 5-year PFS in patients treated with suppressive levothyroxine doses (mean [SD] thyrotropin level, 0.07 [0.13] mIU/L) compared with patients with physiologic levothyroxine doses (mean [SD] thyrotropin level, 3.2 [1.7] mIU/L).^23^ The study did not reveal any differences in DSS between the groups. However, its results might not be easily translatable to ethnically different US or European populations.

The only meta-analysis^9^ published to date summarized the results of 10 heterogeneous case series and cohort studies^10,11,12,13,14,15,16,17,18,19^ involving more than 4000 patients with unknown baseline risk of recurrence. These studies showed a significant 27% risk reduction of combined mortality and disease recurrence rate in patients treated with thyrotropin suppression (eTable 1 in the Supplement).^9^ However, because this meta-analysis did not include other key factors associated with the outcome such as age, disease stage, extent of surgery, and RAI therapy in a logistic regression model and was based on studies published before 1998, prior to current management guidelines, this result may no longer be relevant.

There are 3 studies addressing the association of thyrotropin suppression with mortality and recurrence rate using the National Thyroid Cancer Treatment Cooperative Study Group (NTCTCS) Registry.^12,21,26^ The first study followed 683 patients with DTC (226 with stage III or IV disease) for a median of 4.5 years, documenting that disease stage, patient age, and RAI therapy were associated with disease progression. Thyrotropin level was independently associated with disease progression in patients with high-risk DTC in univariate models (*P* = .03), but not in multivariate models (*P* = .09) (eTable 1 in the Supplement).^12^ The second study involved a larger cohort of 1548 patients with DTC, revealing that OS improved significantly when thyrotropin was suppressed to undetectable levels in 449 patients with stage III or IV disease.^21^ However, DSS in patients with stage III or IV disease was significantly associated only with RAI therapy, while thyrotropin suppression was not independently associated with DSS (eTable 1 in the Supplement).^21^ The third study, using the same NTCTCS database with 3238 patients (939 with stage III or IV disease), revealed that lower initial stage and moderate suppression or low-normal thyrotropin level (ie, not full aggressive suppression) were independently associated with improved 1- to 3-year OS and PFS (eTable 1 in the Supplement).^26^ These results are concordant with our findings of no added benefit of aggressive thyrotropin suppression. All the NTCTCS-based studies analyzed fewer thyrotropin measurements per patient (2-6), while our study involved a mean of 11 thyrotropin measurements per patient, enabling us to draw more accurate conclusions. Reported studies are associated with a significant bias due to performing time-to-event analyses comparing the groups on the basis of future exposures. We have reduced this problem in our study by reporting the landmark analysis.

Last, our study was focused on patients with intermediate- and high-risk DTC treated uniformly with total thyroidectomy and RAI, while the other studies included more heterogeneous groups characterized by a variable baseline risk for recurrence and variable extent of surgery and requirements for RAI. Therefore, evaluating the role of thyrotropin suppression in these groups was more challenging owing to the competing risks.

Another study looking solely at a homogeneous group of patients with high-risk DTC with distant metastases revealed that nonsuppressed thyrotropin and free triiodothyronine greater than 7 pmol/L are independently associated with worse DSS.^28^ The latter is particularly interesting since there are several studies documenting mitogenic effects of triiodothyronine on cancer cell proliferation. Perri et al^31^ demonstrated that triiodothyronine induced proliferation in papillary thyroid carcinoma cell lines via upregulation of cyclin D1 expression. Lin et al^32^ documented that thyroid hormone via αvβ3 is a MAPK-dependent growth factor for thyroid cancer cells in vitro.

Most important, the adverse effects of thyrotropin suppression with supraphysiologic doses of levothyroxine may include exacerbation of angina in patients with ischemic heart disease, increased risk for atrial fibrillation in older patients, and increased risk of osteoporosis in postmenopausal women.^33^

### Limitations

Our study has several limitations, including bias associated with its retrospective design and missing information regarding baseline characteristics in some patients. Although demographic characteristics of the study cohort are consistent with reported characteristics in larger populations,^34^ the results of our study may not apply to the youngest cohort of patients with thyroid cancer younger than 21 years. This group is characterized by a robust expression of sodium iodine symporter in cancer cells and excellent response to therapy with RAI. Thyrotropin receptor expression in younger patients with thyroid cancer might be higher than in older patients, and therefore response to thyrotropin suppression could be different than in the analyzed cohort. While our study is appropriately powered to detect a difference in PFS between groups, it is underpowered in terms of the analysis of factors associated with OS.

## Conclusions

Thyrotropin suppression was not associated with improved PFS in ATA intermediate- and high-risk DTC. A large prospective trial randomizing patients with intermediate- and high-risk DTC to a thyrotropin goal of less than 0.1 mIU/L vs a thyrotropin goal of 0.1 to 0.5 mIU/L and a thyrotropin goal of 0.5 to 2 mIU/L, with regular longitudinal follow-up, is necessary to formulate an unbiased recommendation regarding the optimal thyrotropin goal.

## Notes

Supplement. **eTable 1.** The Effect of Thyrotropin (TSH) Suppression on Overall Survival (OS) and Disease-Specific Survival (DSS) in Intermediate and High-Risk Thyroid Cancer Patients **eTable 2.** Implementation of TSH Scoring System Based on an Example of One of the Enrolled Patients **eTable 3.** Proportionality Assumption for the Landmark Analysis at 1.5, 3 and 5 Years **eTable 4.** Proportion of Patients Assigned to Each TSH Group at Each Landmark **eFigure 1.** The Overview of the Enrollment of Study Participant and Groups Assignment Based on TSH Level **eFigure 2.** No Difference in Progression Free Survival (PFS) in Patients With Different TSH Levels Over Time at Landmark 1.5 (A), 3 (B) and 5 (C) Years **eFigure 3.** No Difference in Overall Survival (OS) in Patients With Different TSH Levels Over Time at Landmark 1.5 (A), 3 (B) and 5 (C) Years
