# Supplementary material for: Association of Thyrotropin Suppression With Survival Outcomes in Patients With Intermediate- and High-Risk Differentiated Thyroid Cancer
Source: JAMA Netw Open. 2019 Feb 1;2(2):e187754. doi: 10.1001/jamanetworkopen.2018.7754 (PMC6484595; doi:10.1001/jamanetworkopen.2018.7754)
Supplement: Supplement. — eTable 1. The Effect of Thyrotropin (TSH) Suppression on Overall Survival (OS) and Disease-Specific Survival (DSS) in Intermediate and High-Risk Thyroid Cancer Patients eTable 2. Implementation of TSH Scoring System Based on an Example of One of the Enrolled Patients eTable 3. Proportionality Assumption for the Landmark Analysis at 1.5, 3 and 5 Years eTable 4. Proportion of Patients Assigned to Each TSH Group at Each Landmark eFigure 1. The Overview of the Enrollment of Study Participant and Groups Assignment Based on TSH Level eFigure 2. No Difference in Progression Free Survival (PFS) in Patients With Different TSH Levels Over Time at Landmark 1.5 (A), 3 (B) and 5 (C) Years eFigure 3. No Difference in Overall Survival (OS) in Patients With Different TSH Levels Over Time at Landmark 1.5 (A), 3 (B) and 5 (C) Years [file jamanetwopen-2-e187754-s001.pdf]

Supplementary Online Content

Klubo-Gwiezdzinska J, Auh S, Gershengorn M, et al. Association of thyrotropin suppression with survival outcomes in patients with intermediate- and high-risk differentiated thyroid cancer. *JAMA Netw Open*. 2019;2(2):e187754. doi:10.1001/jamanetworkopen.2018.7754

eTable 1. The Effect of Thyrotropin (TSH) Suppression on Overall Survival (OS) and Disease-Specific Survival (DSS) in Intermediate and High-Risk Thyroid Cancer Patients

eTable 2. Implementation of TSH Scoring System Based on an Example of One of the Enrolled Patients

eTable 3. Proportionality Assumption for the Landmark Analysis at 1.5, 3 and 5 Years

eTable 4. Proportion of Patients Assigned to Each TSH Group at Each Landmark

eFigure 1. The Overview of the Enrollment of Study Participant and Groups Assignment Based on TSH Level

eFigure 2. No Difference in Progression Free Survival (PFS) in Patients With Different TSH Levels Over Time at Landmark 1.5 (A), 3 (B) and 5 (C) Years

eFigure 3. No Difference in Overall Survival (OS) in Patients With Different TSH Levels Over Time at Landmark 1.5 (A), 3 (B) and 5 (C) Years

This supplementary material has been provided by the authors to give readers additional information about their work.

© 2019 Klubo-Gwiezdzinska J et al. *JAMA Network Open.*

eTable 1. The effect of thyrotropin (TSH) suppression on overall survival (OS) and disease-specific survival (DSS) in intermediate and high-risk thyroid cancer patients.

Study	Number of intermediate/high risk* patients enrolled	Primary outcome	Association between TSH and primary outcome	Average number of TSH measurements	Duration of follow up
**RANDOMIZED CONTROLLED PROSPECTIVE TRIAL**					
Sugitani et al [23]	296 – LN mets<br>50 – AMES high risk**	5 years DSS,<br>5 years PFS	No difference in TSH-S vs TSH-N group<br>5-year PFS 91% vs 89%, p=0.39<br>5-year DSS 99% vs 98%, p=0.31	15	6.9 years
**METAANALYSIS**					
McGriff et al. [9]	No baseline risk data<br>Total number of patients 4174	Combined disease progression and death	TSH (RR 0.73; Cl = 0.60-0.88; P < 0.05), no multivariate model performed	No data	4.5-19.5
**RETROSPECTIVE COHORT STUDIES INCLUDING ALL STAGES OF THYROID CANCER**					
Cooper et al. [12]	229 patients with stage III, IV disease	PFS	Stage (p<0.001), age (p<0.04), RAI therapy (p=0.01);<br>TSH score non-significant in multivariate model (p=0.7)<br>Among high risk patients, treatment with RAI significant (p<0.001), TSH score non-significant (p=0.09)	2.6	4.5 years
Pujol et al. [11]	47 patients with stage III and IV disease	PFS	Advanced stage (HR 2.1, CI 1.1-4.2, p<0.01), non-suppressed TSH (HR 3.2, CI 1.2-8.6, p=0.02), age >45 (HR 2.5, CI 0.9-6.5 p=0.05)	No data	No data
Jonklaas et al. [21]	449 patients with stage III/IV disease with TSH levels available	OS, DSS, PFS	OS for stage III/IV, multivariate model<br>no RAI-tx (HR 1.65 CI 1.21-2.21, p=0.02); non-suppressed TSH (HR 1.9, CI 1.1-3.4, p=0.03)<br><br>DSS for stage III/IV, multivariate model<br>No RAI-tx (HR 1.8 CI 1.2-2.7, p=0.01)	2.3	3 years

© 2019 Klubo-Gwiezdzinska J et al. *JAMA Network Open.*

			Non-suppressed TSH (HR 2.02, CI 0.9-4.8, p=0.09)<br>PFS for stage III/IV, only univariate model available<br>Non-suppressed TSH (HR 1.3, CI 0.8-2.1, p=0.35)		
Hovens et al. [24]	Unknown	DSS, PFS	Multivariate model for DSS<br>For Stages T1-3 M0 median TSH (HR 2.14, CI 1.18 –3.89, p=0.01)<br>TSH>2 mIU/ml associated with decreased DSS<br>Multivariate model for PFS<br>For Stages T1-3 M0 median TSH (HR 1.41 CI 1.03–1.95, p=0.03)<br>TSH>2 mIU/ml associated with decreased PFS	>4	8.8 years
Carhill et al.[26]	939 stage III/IV patients	OS, PFS	Multivariate model for OS<br>Stage III vs II (HR 5.3 CI 3.5-8.4, p<0.001)<br>Stage IV vs III (HR 3.3 CI 2.4-4.6, p<0.001)<br>Mean TSH score 2-2.9 vs 3-4 (HR 0.17 CI 0.12-0.3, p<0.001)<br>Mean TSH score 1-1.9 vs 2-2.9 (HR 0.95, CI 0.7-1.3, p=0.8)<br>Multivariate model for PFS<br>Stage III vs II (HR 2.1, CI 1.6-2.7, p<0.001)<br>Mean TSH score 2-2.9 vs 3-4 (HR 0.3 CI 0.2-0.4, p<0.001)<br>Mean TSH score 1-1.9 vs 2-2.9 (HR 1.3, CI 0.98-1.6, p=0.07)	6	6 years

© 2019 Klubo-Gwiezdzinska J et al. *JAMA Network Open.*

RETROSPECTIVE COHORT STUDIES FOCUSED ON PATIENTS WITH DISTANT METASTASES					
Diessl et al. [28]	157 patients with distant metastases	DSS	Higher mean TSH >0.1 (p=0.001) and median free T3 >7 pmol/l (p=0.01) independently worsening DSS	>3	8 years

*****Intermediate/high risk patients – patients with tumors larger than 4 cm and/or with microscopic or gross extrathyroidal extension, and/or with lymph nodes or distant metastases

** **AMES High-risk** – patients with distant metastases; older patients with major capsular involvement papillary cancer or major capsular involvement follicular carcinoma and tumor size ≥5

DSS – disease specific survival, OS – overall survival, PFS – progression-free survival, LN – lymph nodes, TSH-S – TSH suppressed, TSH-N – TSH normal, RR – relative risk, RAI – radioactive iodine, RAI-tx – treatment with RAI, HR- hazard ratio

© 2019 Klubo-Gwiezdzinska J et al. *JAMA Network Open.*

eTable 2. Implementation of TSH scoring system based on an example of one of the enrolled patients.

Patient's identifier #	Date of TSH	TSH value [mIU/ml]	TSH score	Clinical comment
NIH2	9/7/2006	0.05	1	
NIH2	1/9/2007	0.05	1	
**NIH2**	**2/15/2007**	**82.7**	**4**	**STIMULATION**
NIH2	5/29/2007	0.13	2	
NIH2	8/28/2007	0.24	2	
NIH2	12/3/2007	0.14	2	
NIH2	1/29/2008	0.02	1	
NIH2	8/5/2008	<0.02	1	
NIH2	12/30/2008	0.02	1	
NIH2	4/13/2009	<0.02	1	
**NIH2**	**4/17/2009**	**15.1**	**4**	**STIMULATION**
NIH2	9/21/2010	0.02	1	
NIH2	10/29/2010	0.02	1	
**NIH2**	**12/17/2010**	**54.7**	**4**	**STIMULATION**
NIH2	3/1/2011	0.04	1	
NIH2	3/15/2011	0.13	2	
NIH2	6/28/2011	0.02	1	
NIH2	7/22/2011	0.03	1	
NIH2	9/6/2011	0.05	1	
NIH2	10/23/2012	< 0.01	1	
NIH2	2/5/2013 13:57	< 0.01	1	
NIH2	9/10/2013	< 0.001	1	
NIH2	4/7/2014 16:50	0.01	1	
**NIH2**	**4/9/2014 12:30**	**119.8**	**4**	**STIMULATION**
NIH2	7/14/2015	< 0.01	1	
**AVERAGE**		**14.38263**	**1.64**	**TSH-S GROUP**

© 2019 Klubo-Gwiezdzinska J et al. *JAMA Network Open.*

eTable 3. Proportionality assumption for the landmark analysis at 1.5, 3 and 5 years.

	P value PFS	P value OS
**Landmark 1.5 year**	0.62	0.50
**Landmark 3 years**	0.70	0.69
**Landmark 5 years**	0.41	0.30

eTable 4. Proportion of patients assigned to each TSH group at each landmark.

TSH-S – patients with longitudinally suppressed TSH (score 1-<2), TSH-ML – patients with longitudinally moderately suppressed/low normal TSH (score 2-<3), TSH-LE – patient with longitudinally low normal/elevated TSH (score 3-4)

Proportion of patients assigned to TSH-S group (average score 1-<2)	Proportion of patients assigned to TSH-ML group (average score 2-<3)	Proportion of patients assigned to TSH-LE group (average score 3-4)
**OVERALL N=867 PATIENTS**		
339 (39.1%)	328 (37.8%)	200 (23.1%)
**LANDMARK 1.5 YEARS N=591**		
143 (24.2%)	205 (34.7%)	243 (41.1%)
**LANDMARK 3 YEARS N=670**		
204 (30.4%)	266 (39.7%)	200 (29.9%)
**LANDMARK 5 YEARS N=755**		
277 (36.7%)	293 (38.8%)	185 (24.5%)

© 2019 Klubo-Gwiezdzinska J et al. *JAMA Network Open.*

eFigure 1. The overview of the enrollment of study participant and groups assignment based on TSH level. A) Study population B) Group assignment based on TSH scoring system. TSH-S – patients with suppressed TSH during follow up, TSH-ML – patients with moderately suppressed/low normal TSH over time, TSH-LE – patient with low normal/elevated TSH during follow up.

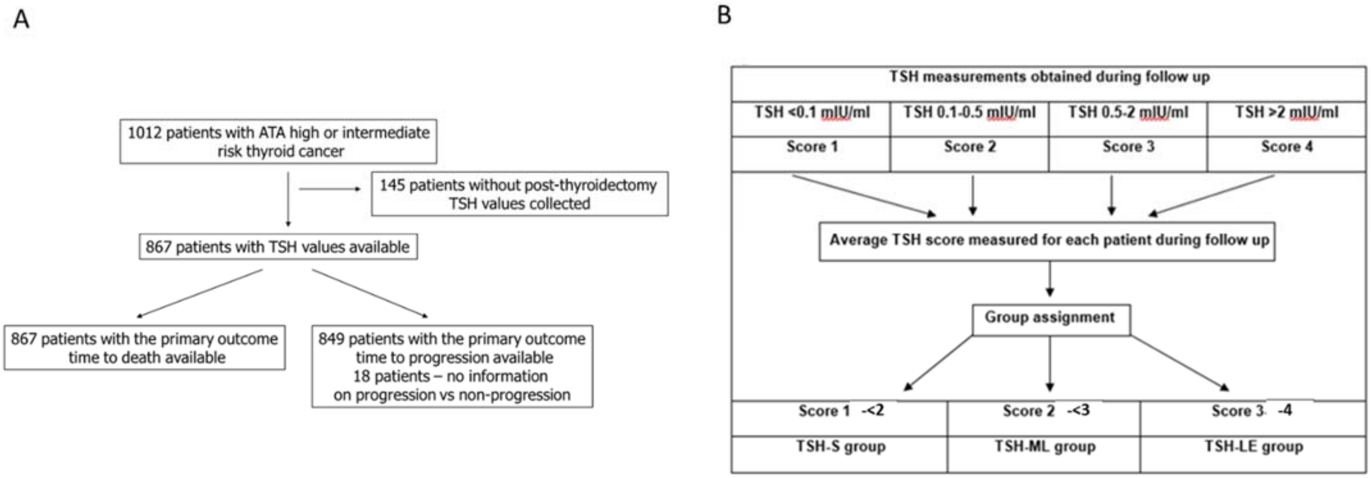

© 2019 Klubo-Gwiezdzinska J et al. *JAMA Network Open.*

eFigure 2 No difference in progression free survival (PFS) in patients with different TSH levels over time at landmark 1.5 (A), 3 (B) and 5 (C) years. TSH-S – patients with suppressed TSH (score 1-<2), TSH-ML – patients with moderately suppressed/low normal TSH (score 2-<3), TSH-LE – patient with low normal/elevated TSH (score 3-4).

A

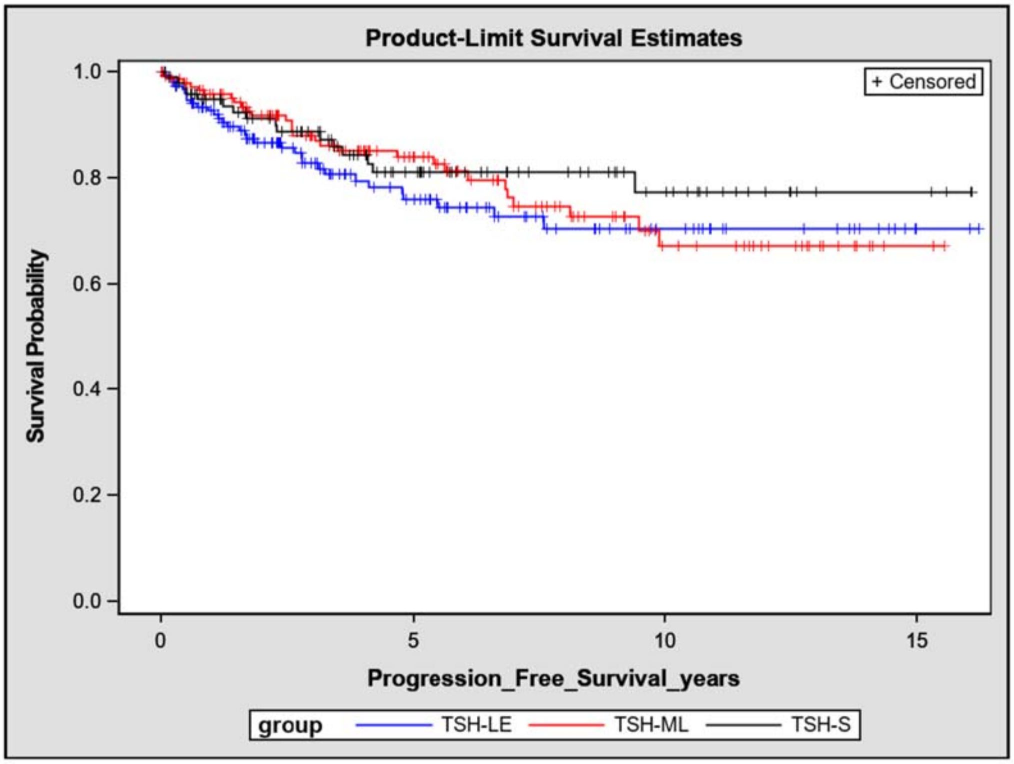

B

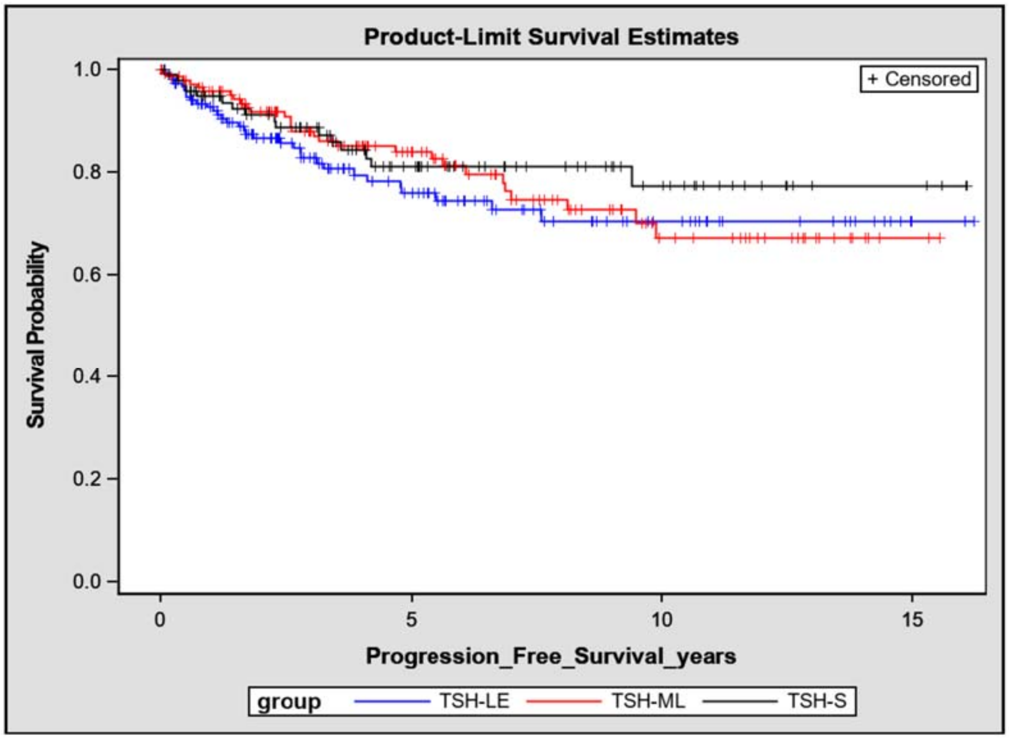

© 2019 Klubo-Gwiezdzinska J et al. *JAMA Network Open.*

C

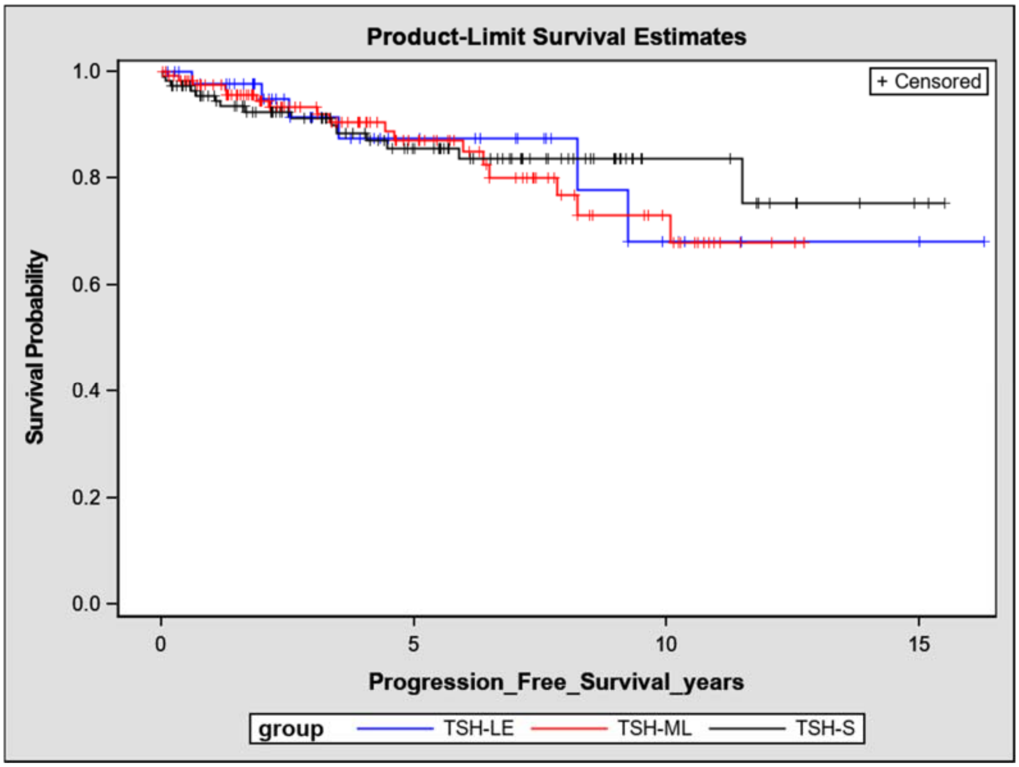

© 2019 Klubo-Gwiezdzinska J et al. *JAMA Network Open.*

eFigure 3 No difference in overall survival (OS) in patients with different TSH levels over time at landmark 1.5 (A), 3 (B) and 5 (C) years. TSH-S – patients with suppressed TSH (score 1-<2), TSH-ML – patients with moderately suppressed/low normal TSH (score 2-<3), TSH-LE – patient with longitudinally low normal/elevated TSH (score 3-4)

A

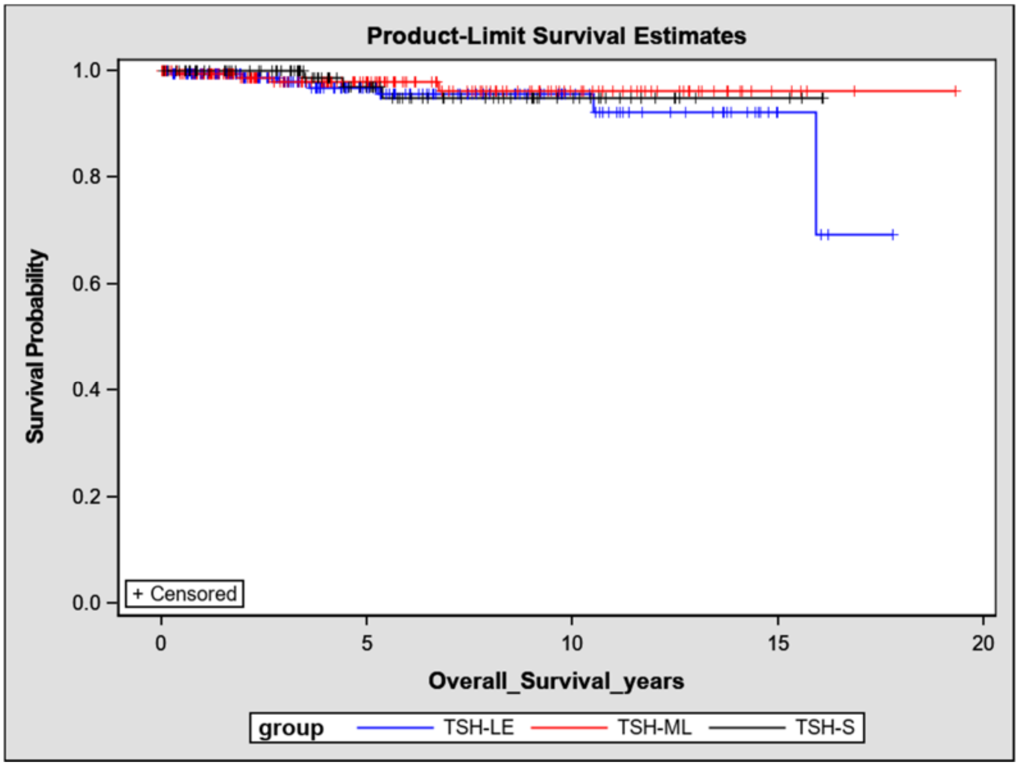

B

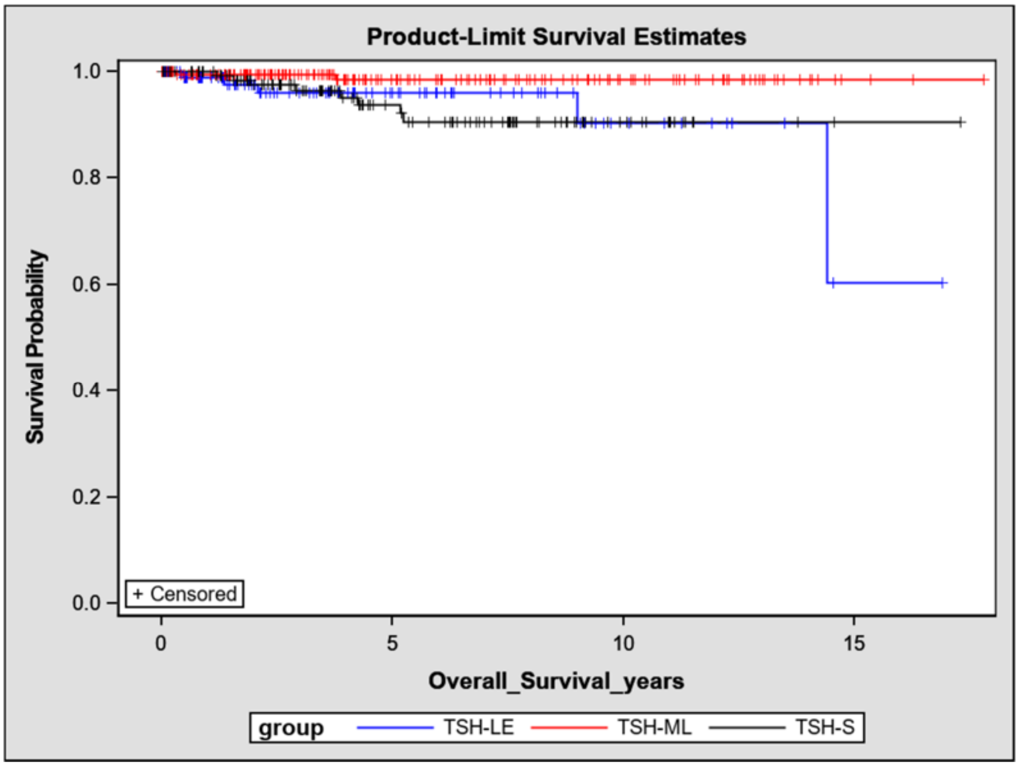

© 2019 Klubo-Gwiezdzinska J et al. *JAMA Network Open.*

C

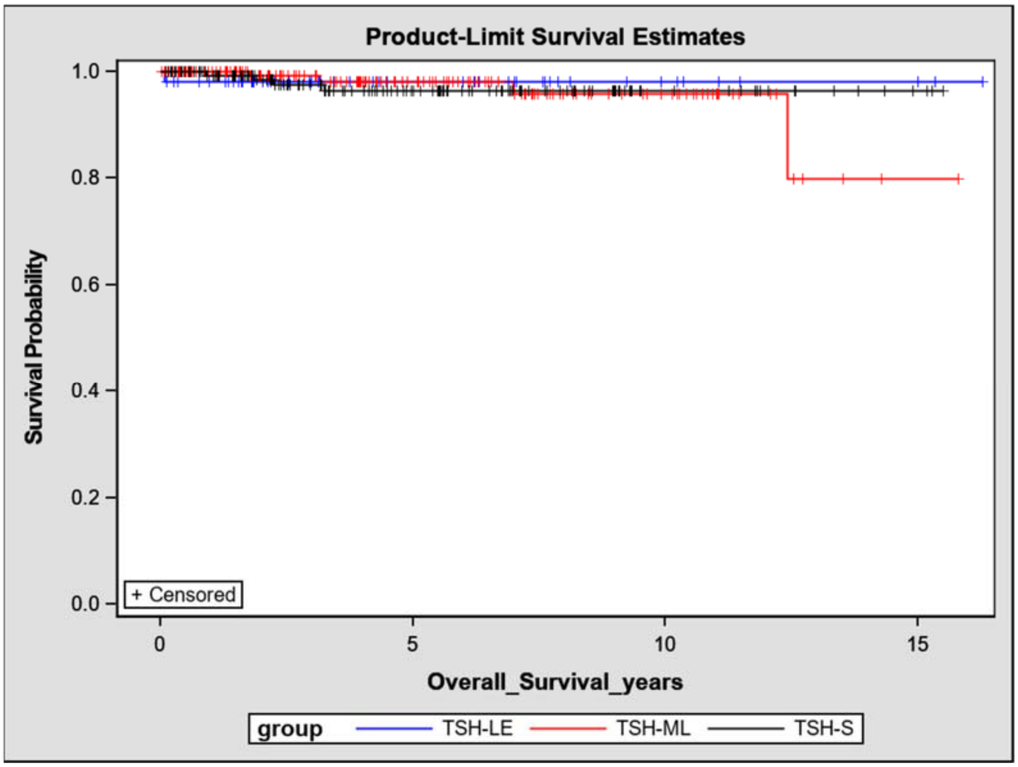

© 2019 Klubo-Gwiezdzinska J et al. *JAMA Network Open.*
